# The association between use of benzodiazepines and risk of suicide: A systematic review

**DOI:** 10.1192/j.eurpsy.2025.2365

**Published:** 2025-08-26

**Authors:** M. Oliveró, M. Martínez García, V. Pérez, M. T. Campillo, G. Martínez-Alés, L. Cano, D. Guinart, P. Mortier

**Affiliations:** 1 Psychiatry, Hospital del Mar; 2 Psychiatry and forensic medicine, Universidad Autónoma; 3 Medical Director Hospital de Mar and Mental Health Institute; 4 Neurosciences Research Unit, Hospital del Mar d’Investigacions Mèdiques; 5 Department of Experimental and Health Sciences, Pompeu Fabra University; 6 Centro de Investigación Biomédica en Red de Salud Mental (CIBERSAM G21), Instituto de Salud Carlos III, Barcelona, Spain; 7 Department of Psychiatry, Icahn School of Medicine at Mount Sinai, New York; 8 CAUSALab, Harvard TH Chan School of Public Health, Boston, United States; 9 Hospital La Paz Institute for Health Research (IDIPaz); 10 Mental Health Network Biomedical Research Center (CIBERSAM), Madrid; 11 Hospital del Mar Research Institute, CIBERSAM, Barcelona, Spain; 12 The Donald and Barbara Zucker School of Medicine at Hofstra/Northwell, New York, United States; 13 Health Services Research Group, Hospital del Mar Research Institute; 14 Centro de Investigación Biomédica en Red de Epidemiología y Salud Pública, Instituto de Salud Carlos III, Barcelona, Spain

## Abstract

**Introduction:**

Benzodiazepines are widely prescribed for the management of common mental health disorders. Although a direct relationship between benzodiazepines and suicide risk reduction has not been described, they can be an adequate choice to palliate anxiety and insomnia in patients with suicidal ideation. This is of special interest considering that both anxiety and insomnia are risk factors for suicidal behaviours themselves (May and Klonsky. Clin Psychol: Science and Practice. 2016; 23 5–20)(Park *et al.* J Psychiatr Res. 2020; 131 1-8). Nonetheless, paradoxically, there is a rising concern regarding an increase in suicide risk associated to benzodiazepine use, as some recent evidence seems to suggest (Dodds. Prim Care Companion CNS Disord 2017; 2;19)(McCall *et al.* Am J Psychiatry 2017; 1;174 18-25). Clarifying this potential association can help guide clinical decision-making to promote suicide prevention.

**Objectives:**

To review the currently available evidence regarding the relationship between the use of benzodiazepines for common mental disorders and subsequent suicide, suicidal behaviours and self-injurious behaviours.

**Methods:**

A systematic review of the literature was conducted using a combination of search terms related to “suicide” and “benzodiazepine” to assess publications from inception to February 2024 in 3 different databases (Scopus, PsychInfo, MEDLINE). Eligibility criteria included experimental, observational studies and previous systematic reviews while excluding conference proceedings, case reports/series, editorials, opinion papers and letters. Studies involving individuals with severe psychiatric disorders, dementia or personality disorder were also excluded. Risk of bias was assessed in RCTs with the RoB 2 tool while EPHPP Assessment Tool 2010 was applied for other study types.

**Results:**

A total of 2090 titles and abstracts were screened; 19 papers were reviewed for inclusion and 8 were included in this review for data extraction. Most of the included publications consisted of observational studies. Results tended to indicate a higher risk of suicide, suicidal behaviour and/or self-injurious behaviours in relation to benzodiazepine use although data was contradictory and affected by confounding.

**Conclusions:**

Evidence seems to suggest a positive relationship between benzodiazepines and suicide, suicidal behaviour and/or self-injurious behaviours although, due to the predominant observational study designs and the presence of unadjusted confounding, these results must be extrapolated with care and no causality can be inferred. An interesting approach for future research to palliate such limitations could be Target Trial Emulation (Hernán and Robins. Am J Epidemiol 2016; 15;183(8) 758-64), which has already been adopted to guide decision-making in absence of randomized trials in many fields of medicine.

**Disclosure of Interest:**

M. Oliveró: None Declared, M. Martínez García: None Declared, V. Pérez Grant / Research support from: Has received honoraria or grants from AB-Biotics, AstraZeneca, Bristol-Myers-Squibb, CIBERSAM, FIS- ISCiii, Janssen Cilag, Lundbeck, Otsuka, Servier and Medtronic., Consultant of: Has been a consultant to AB-Biotics, AstraZeneca, Bristol-Myers-Squibb, CIBERSAM, FIS- ISCiii, Janssen Cilag, Lundbeck, Otsuka, Servier and Medtronic., M. T. Campillo: None Declared, G. Martínez-Alés Grant / Research support from: American Foundation for Suicide Prevention grant ECR-1-101-23, Brain and Behavior Research Foundation grant NARSAD 31312 and National Institutes of Mental Health grant 1R25MH129256-01A1, L. Cano: None Declared, D. Guinart Grant / Research support from: CM21/00033, Consultant of: Has been a consultant and/or advisor or has received honoraria from: Angelini, Otsuka, Lundbeck and Teva., P. Mortier Grant / Research support from: This work was supported by grant CP21/00078 from the ISCIII-FSE Miguel Servet

